# Long-term exposure to ambient fine particulate matter chemical composition and in-hospital case fatality among patients with stroke in China

**DOI:** 10.1016/j.lanwpc.2022.100679

**Published:** 2023-01-13

**Authors:** Miao Cai, Xiaojun Lin, Xiaojie Wang, Shiyu Zhang, Chongjian Wang, Zilong Zhang, Jay Pan, Hualiang Lin

**Affiliations:** a Department of Epidemiology, School of Public Health, Sun Yat-sen University, NO. 74, Zhongshan 2nd Road, Yuexiu District, Guangzhou, Guangdong, 510080, China; b HEOA Group, West China School of Public Health and West China Fourth Hospital, Sichuan University, No. 16, Section 3, Ren Min Nan Road, Chengdu, Sichuan, 610041, China; c Institute for Healthy Cities and West China Research Center for Rural Health Development, Sichuan University, No. 16, Section 3, Ren Min Nan Road, Chengdu, Sichuan, 610041, China; d Department of Epidemiology and Biostatistics, College of Public Health, Zhengzhou University, Zhengzhou, Henan, 450001, China; e School of Public Administration, Sichuan University, No.24 South Section I, Yihuan Road, Chengdu, Sichuan, 610065, China

**Keywords:** Fine particulate matter, Chemical components, Stroke, In-hospital mortality, China

## Abstract

**Background:**

There is little evidence on the association between PM_2.5_ chemical components and fatality among hospitalized stroke patients.

**Methods:**

This study used an inpatient discharge database from 2013 to 2019 in four provinces (Sichuan, Shanxi, Guangxi, and Guangdong) in China. Annual average exposure to PM_2.5_ and its five chemical components [black carbon (BC), organic matter (OM), sulphate (${SO}_{4}^{2-}$), nitrate (${NO}_{3}^{-}$), and ammonium (${NH}_{4}^{+}$)] were estimated using bilinear interpolation at patient's residential address. Mixed-effects logistic regression models were conducted to estimate the odds ratios (ORs). Counterfactual analyses were used to estimate the population attributable burden (PAF).

**Findings:**

Among 3,069,093 hospitalized patients with stroke, each interquartile (IQR) increment in PM_2.5_ and its chemical components was significantly associated with stroke fatality: the ORs were 1.137 [95% confidence interval (CI): 1.118–1.157; IQR: 15.14 μg/m^3^] for PM_2.5_, 1.108 (95% CI: 1.091–1.126; IQR: 0.71 μg/m^3^) for BC, 1.086 (95% CI: 1.069–1.104; IQR: 3.47 μg/m^3^) for OM, and 1.065 (95% CI: 1.048–1.083; IQR: 2.81 μg/m^3^) for ${SO}_{4}^{2-}$. We did not find significant associations for ${NO}_{3}^{-}$ (OR: 0.991, 95% CI: 0.975–1.008; IQR: 3.30 μg/m^3^). The associations were larger among patients with ischemic stroke than those with hemorrhagic stroke. The PAFs were 10.6% (95% CI: 9.1–12.2%) for BC, 9.9% (95% CI: 8.2–11.7%) for OM, and 6.6% (4.9–8.3%) for ${SO}_{4}^{2-}$.

**Interpretation:**

Ambient BC, OM, and ${SO}_{4}^{2-}$ might be important risk factors for stroke fatality. The findings advocate the need to develop tailored guidelines for PM chemical components and curb the emissions of the most hazardous chemical components.

**Funding:**

10.13039/100000865Bill & Melinda Gates Foundation (INV-016826).

**Research in context**

**Evidence before this study**

Ambient PM_2.5_ is the second leading cause of disability-adjusted life year among those with stroke in East Asia. Abundant studies have reported the associations of ambient fine particulate matter (PM_2.5_) with stroke mortality. We searched PubMed, Web of Science, and Google Scholar using the terms “fine particulate matter”, “PM_2.5_”, “chemical components”, “chemical composition”, “chemical constituents”, “death”, “mortality”, and “stroke” in English for studies published up to August 24, 2022. However, we did not find studies that examined the relationship between chemical components of PM_2.5_ and mortality among patients hospitalized with stroke.

**Added value of this study**

This is the largest study that investigated the associations between long-term exposure to chemical components of PM_2.5_ and stroke mortality. By analyzing the data of over 3 million hospitalized patients in 4 provinces of China, we found that each interquartile range increment in annual concentrations of black carbon [odds ratio (OR): 1.137, 95% confidence interval (CI): 1.118–1.157], organic matter (OR: 1.086, 95% CI: 1.069–1.104), and sulfate (OR: 1.065, 95% CI: 1.048–1.083) at residential addresses were associated with the highest risk of fatality in hospitalized stroke patients.

**Implications of all the available evidence**

This study identifies black carbon, organic matter, and sulfate, mostly from anthropogenic and combustion-related sources, as the most hazardous chemical components to stroke mortality. The results highlight the need to establish finer ambient air quality guidelines and formulate more targeted regulations on PM_2.5_ chemical components.

## Introduction

Stroke was the second-leading cause of death globally in 2019.^1^ Approximately 87% of global stroke-related deaths and disability-adjusted life years occur in low- and middle-income countries (LMICs).^2^ In China, stroke led to 2.19 million deaths in 2019 and mortality rate has increased by 32.3% in the recent 30 years.^3^ The burden of stroke is determined by a constellation of modifiable factors including metabolic, behavioral, and environmental risk factors.^1^

Ambient particulate matter (PM) air pollution was the fourth-leading cause of stroke-related deaths and disabilities globally in 2019, and it was the second-leading cause of stroke-related disability-adjusted life year in East Asia.^1^ Numerous studies have shown that ambient PM_2.5_ (fine particulate matter ≤2.5 μm in aerodynamic diameter) air pollution is associated with an increased risk of stroke incidence, hospitalization, and mortality.^4^, ^5^, ^6^, ^7^, ^8^ However, these studies have been limited to evaluating PM mass as a whole, failing to assess the toxicity of each PM chemical component. Given the differences in chemical properties of each PM chemical component, it is unlikely that they have equally important adverse health effects.^9^ The World Health Organization (WHO) published the most up-to-date air quality guideline in 2021,^10^ but no guidelines have yet been established for the chemical compositions of PM_2.5_. Characterizing the relationships between PM chemical composition and stroke mortality could help expand the existing WHO air quality guideline to PM_2.5_ chemical components and their emission sources, as well as promote more targeted regulations and policies to alleviate the health burden attributable to PM air pollution. In China, where the majority of residents experience poor air quality, stroke is the leading cause of death and long-term disability.^11^ Patients with stroke are often hospitalized to receive timely administration of thrombolytic therapy and endovascular surgery, as well as targeted critical care,^12^ but there is a lack of studies that investigates the relationship between PM_2.5_ chemical components and mortality among patients hospitalized with stroke. Evidence on the link between PM_2.5_ chemical components and fatality among patients hospitalized with stroke from China may illuminate policy formulation in other LMICs, which comprise much of the world's population and bear an exceedingly high burden of stroke and air pollution.

In this study, we used data from a large volume of hospitalized patients from a multi-province inpatient hospitalization database in China (N > 3 million). We then assessed individual-level exposure to ambient PM_2.5_ and chemical components in their residential addresses before hospitalization. We subsequently characterized the relationship between PM_2.5_ chemical components [black carbon (BC), organic matter (OM), sulphate (${SO}_{4}^{2-}$), nitrate (${NO}_{3}^{-}$), and ammonium (${NH}_{4}^{+}$)] and stroke fatality.

## Methods

### Data collection

**Fig. 1:**
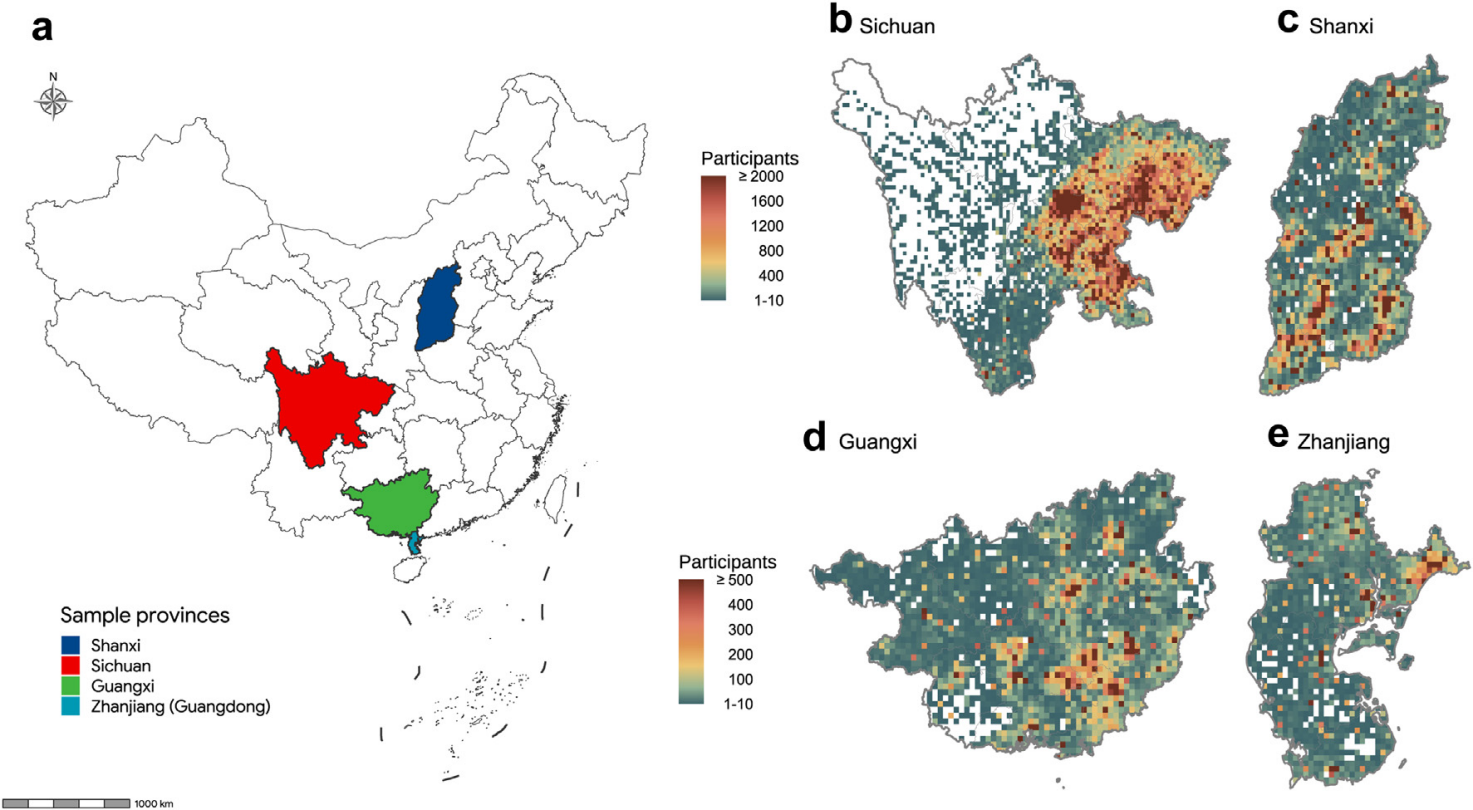
Spatial distribution of sample provinces (1a.) and patients' residential addresses in four Chinese provinces (1b-1e).

Using the inpatient discharge database in four provinces in China,^13^, ^14^, ^15^ we collected individual-level data on patient demographics, medical diagnoses and procedures, residential addresses, and in-hospital health outcomes from Sichuan, Shanxi, Guangxi, and Guangdong (Zhanjiang city) (Fig. 1a). The ranges of data collection dates were: from January 1, 2018, to December 31, 2019, for Sichuan; from January 1, 2013, to December 31, 2018, for Shanxi; and from January 1, 2013, to December 31, 2016, for Guangxi and Zhanjiang city. Patient identifiers including names and unique identification numbers were removed prior to accessing the data. The study was approved by institutional review board of the School of Public Health, Sun Yat-sen University.

### Inclusion and exclusion criteria

**Fig. 2:**
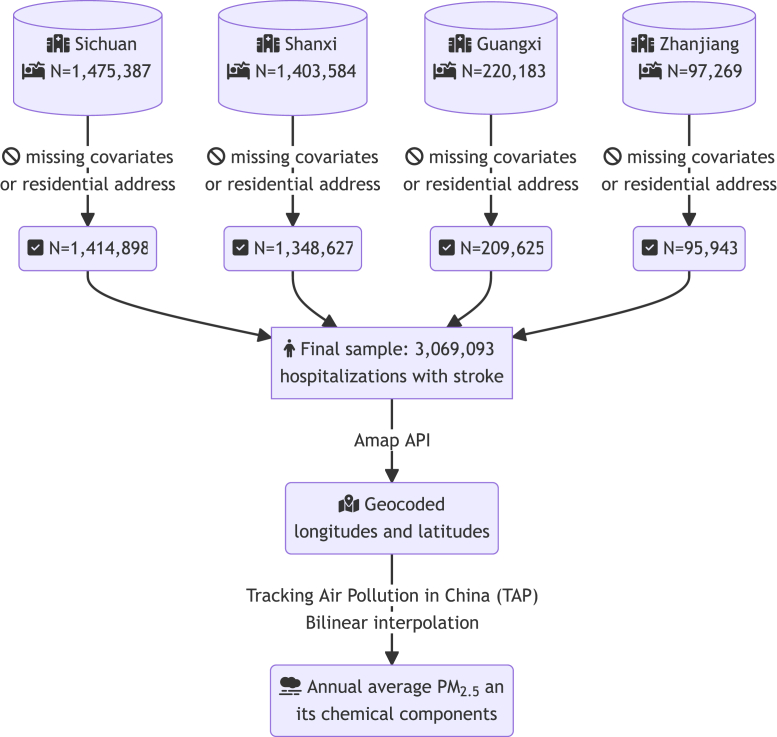
**A flowchart of sample selection, inclusion or exclusion criteria, and exposure measurement**. API: application programming interface; TAP: tracking air pollution in China.

Patients with stroke were identified using the primary clinical diagnosis code (International Classification of Diseases, Tenth Revision, Clinical Modification [ICD-10-CM]) at admission.^16^, ^17^, ^18^ Ischemic stroke was defined as primary diagnosis ICD-10 code containing I63 and H34.1; hemorrhagic stroke was defined as primary diagnosis ICD-10 code containing I60, I61, and G45; and stroke of unspecific type was defined as primary diagnosis ICD-10 code containing I64. We excluded patients who were younger than 18 years old, whose age, sex, ethnicity, and residential addresses were unknown, as well as those who had no data on exposure. A flowchart for sample inclusion and exclusion is shown in Fig. 2.

### Exposure measurement

Daily concentrations of PM_2.5_ mass and its dust-free chemical components (BC, OM, ${SO}_{4}^{2-}$, ${NO}_{3}^{-}$, and ${NH}_{4}^{+}$) at 10 × 10 km spatial resolution were obtained from the Tracking Air Pollution [TAP] database in China (http://tapdata.org.cn/).^19^^,^^20^ TAP PM chemical composition data synthesize situ measurements data from ground-based measurements, satellite-based aerosol optical depth estimates, and bottom-up emission inventory, and estimate their daily concentrations using Weather Research and Forecasting (v3.9.1)-Community Multiscale Air Quality (v5.2) chemical transport models in China from 2000 to present.^20^ The TAP data source for PM_2.5_ mass had an average out-of-bag cross-validation correlation coefficient (R) of 0.83, and the model cross-validation Rs were 0.70 for ${SO}_{4}^{2-}$, 0.75 for ${NH}_{4}^{+}$, 0.72 for OM, 0.64 for BC, and 0.75 for ${NO}_{3}^{-}$, representing overall good consistency with ground measurement of PM chemical components.^19^^,^^20^

**Fig. 3:**
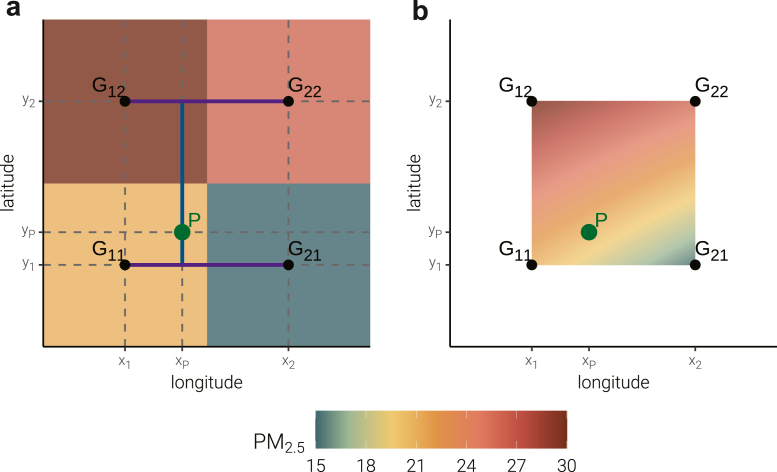
**Bilinear interpolation method to estimate ambient air pollution at patient's residential address (P) using the nearest four grids.** (a) The four colored squares denote the gridded raster data for air pollution, the four black points are their center points, and G_11_, G_12_, G_21_, G_22_ are the concentration of air pollution in each grid. P (longitude: x_P_; latitude y_P_) is the location of patient's residential address. (b) The concentration of air pollution estimated using bilinear interpolation method within the four center points.

Annual concentrations (365-day average) of PM_2.5_ mass and its chemical components, as well as seven-day average levels of temperature and relative humidity, were estimated at patients' residential address prior to the day of hospitalization. Using the application programming interface of amap (also known as Gaode map), latitudes and longitudes of the study patients were obtained by geocoding the patients' residential address prior to hospitalization. We then used bilinear interpolation to estimate the levels of environmental variables prior to hospitalization at patients' resident addresses.^6^^,^^21^ Fig. 3 shows the bilinear interpolation method that estimates environmental variables at patient's residential address P using the nearest four gridded raster data. The concentration of environmental variables at location P is denoted as *G*(p), and it can be estimated as a weighted average of the nearest four grids surrounding a residential address using the following formula:

$$G\left(P\right)=G_{11}\omega_{11}+G_{12}\omega_{12}+G_{21}\omega_{21}+G_{22}\omega_{22}$$

$$\omega_{11}=\frac{\left(x_{2}-x_{P}\right)\left(y_{2}-y_{P}\right)}{\left(x_{2}-x_{1}\right)\left(y_{2}-y_{1}\right)}$$

$$\omega_{12}=\frac{\left(x_{2}-x_{P}\right)\left(y_{P}-y_{1}\right)}{\left(x_{2}-x_{1}\right)\left(y_{2}-y_{1}\right)}$$

$$\omega_{21}=\frac{\left(x_{P}-x_{1}\right)\left(y_{2}-y_{P}\right)}{\left(x_{2}-x_{1}\right)\left(y_{2}-y_{1}\right)}$$

$$\omega_{22}=\frac{\left(x_{P}-x_{1}\right)\left(y_{P}-y_{1}\right)}{\left(x_{2}-x_{1}\right)\left(y_{2}-y_{1}\right)}$$

Where $G_{11},G_{12},G_{21},and\;G_{22}$ are the concentrations of air pollution in the nearest four grids around location P, and $\omega_{11},\omega_{12},\omega_{21},and\;\omega_{22}$ are the associated weights (Fig. 3a). The weights depend on the distance from the residential address to the grids of air pollutants, and the closer the distance from the patient's address to the grid, the larger the weights associated with the grids are. The estimated concentrations using bilinear interpolation method are shown in Fig. 3b. Bilinear interpolation enhances the spatial resolution of exposure measurement by transforming the gridded data into smoothed version of concentrations.

### Outcome

The outcome is all-cause case fatality during hospital stay. The variable was ascertained by the attending physician and obtained from the discharge status field from the inpatient discharge database.

### Covariates

Covariates were selected based on data availability and potential confounding to the associations of PM_2.5_ mass and its chemical components with stroke fatality. Age, sex, and ethnicity (Han and non-Han Chinese) were included as demographic variables; occupation (public sector, private sector, agriculture, unemployed, retired, or other) and marital status (married, single, widowed, divorced, or other) were included to represent socioeconomic status. A set of clinical comorbidities were selected to control for disease severity and potential confounding issues: hypertension, diabetes, congestive heart failure, cardiac arrhythmias, peripheral vascular disorders, and liver disease. These comorbidities were identified by ICD-10 diagnosis codes from up to 15 secondary diagnosis fields and further enhanced by keywords matching in clinical diagnosis text description fields. Besides, we included intracranial procedure as a dummy variable, which was identified using ICD-9 procedure codes and text matching in procedure text description using regular expressions. Hospital level (tertiary and non-tertiary) was included to represent hospital volume, where tertiary hospitals are large medical centers located in urban metropolitan areas.^22^^,^^23^ Daily meteorological data on temperature and relative humidity at 9 × 9 km spatial resolution were obtained from the fifth generation of European ReAnalysis (ERA5)–Land reanalysis data set.^24^ We included seven-day average temperature and relative humidity prior to the day of hospitalization as natural cubic splines with five degrees of freedom. These splines were constructed to capture any potential nonlinear relationships between temperature, relative humidity, and the risk of mortality as previous studies have shown evidence for J-shape or U-shape relationships.^25^

### Statistical analyses

The characteristics of the overall participants and by fatality status were presented as median (interquartile range, IQR) for continuous variables and as frequency (percent) for categorical variables. We constructed mixed-effects logistic regression models to estimate the odds ratios (ORs) per IQR increment in PM_2.5_ mass and the five chemical components and the associated 95% confidence intervals (CIs), where the provinces and cities were considered as random intercepts. To illustrate potential nonlinear relationships and estimate concentration-response curves, we included PM_2.5_ and its chemical components as natural cubic splines and the knots were specified at quartiles.

We estimated PAFs using counterfactual analyses. The potential reducible number of fatalities were calculated as the difference between the observed number of fatalities and predicted number of fatalities in a counterfactual scenario of air pollutants while other covariates remained identical. The counterfactual scenario was set at hypothetically optimal and feasible concentrations (the lower fifth percentiles in their statistical distributions) of PM_2.5_ mass and its chemical components in the study sample, while those observed concentrations lower than the optimal ones were kept unchanged. PAFs were then computed as the division of potential reducible number of fatalities by the observed total number of fatalities. We estimated bootstrap 95% CIs for the PAFs using 1000 replicate samples for each model.

All statistical tests were two-sided, and a 95% CI of an OR that excluded unity or a P-value ≤0.05 was considered statistically significant. All data cleaning, statistical modeling, and data visualization were done using statistical computing environment R 4.1.3. The study was reported according to the Strengthening the Reporting of Observational Studies in Epidemiology (STROBE) reporting guideline.

### Sensitivity analysis

We undertook a set of sensitivity analyses to test the robustness of our findings. (A) To check the consistency of findings to alternative model specifications, we used time to fatality within hospitals as the outcome and estimated the hazard ratios using Cox proportional hazard models. (B) To mitigate potential confounding caused by a limited number of clinical comorbidities, we included Elixhauser comorbidity score as a covariate instead of the six comorbidity dummy variables in a sensitivity analysis. Elixhauser comorbidity score is a weighted average of a more comprehensive list of over 30 comorbidities and showed good prediction accuracy in the Chinese population.^15^ It is one of the best-known indices in the field of health service research. It is widely used to measure disease burden and adjust for patient risk with administrative data. The Elixhauser comorbidities were defined using all secondary diagnosis codes using the coding algorithm defined by Quan et al.^26^ Cardiovascular comorbidities include congestive heart failure and cardiac arrhythmias, while stroke/cerebrovascular disease was not included as a comorbidity in the algorithm. (C) To further examine the issue of unmeasured confounding and examine the evidence for causality, we computed the E-values of the ORs and their 95% CIs in our main models. E-value is the minimum strength of the confounder-exposure and confounder–outcome associations for an unmeasured confounder to neutralize the observed association.^27^, ^28^, ^29^ A larger E-value suggests stronger evidence for causal relationship, and the minimal value is one. (D) We re-estimated the ORs and 95% CIs in a sample with informative data on occupation and marital status by excluding the other category. (E) Since our sample were collected from four different provinces, we further conducted our models in samples stratified by provinces and check the concordance of results by provinces. (F) Since the data were collected in different time periods and may lead to systematic errors, we conducted a sensitivity analysis by restricting to a subsample of unified time range of 825,340 hospitalizations from 2013 to 2016 in Shanxi, Guangxi, and Zhanjiang. (G) To investigate the potential bias caused by patients with multiple admissions, we conducted a sensitivity analysis among sample without previous admissions in Sichuan province. We did not conduct this analysis based on data from Shanxi, Guangxi, and Zhanjiang as we did not have data on unique identification number of the patients in these regions.

### Role of the funding source

The funders had no role in the design and conduct of the study; collection, management, analysis, and interpretation of the data; preparation, review, or approval of the manuscript; and decision to submit the manuscript for publication.

## Results

Among 3,069,093 hospitalized cases with the primary diagnosis of stroke, 31,532 (1.03%) experienced fatality in hospitals. Fig. 1b–e presents choropleth maps in each of the four provinces, showing the geographical distribution of the study participants. Among the participants, 1,414,898 (46.1%) were from Sichuan province, 1,348,627 (43.94%) were from Shanxi province, 209,625 (6.83%) from Guangxi province, and 95,943 (3.12%) from Zhanjiang, Guangdong province.

**Fig. 4:**
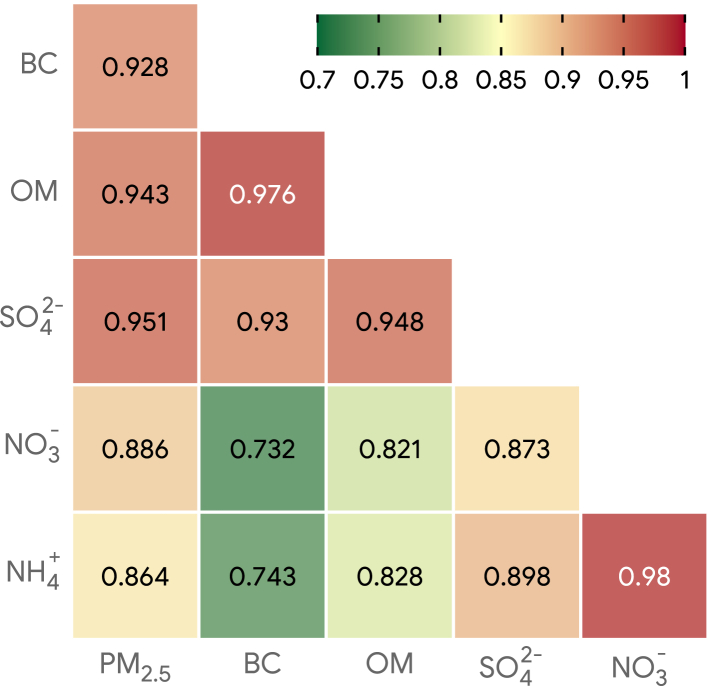
Pairwise Pearson correlation coefficients between annual concentrations of ambient PM_2.5_ and its chemical compositions [black carbon (BC), organic matter (OM), sulphate (${SO}_{4}^{2-}$), nitrate (${NO}_{3}^{-}$), and ammonium (${NH}_{4}^{+}$)] prior to the day of hospitalization.

Among the study sample (median age 68 years and 43.3% females), those who experienced in-hospital fatalities were older, had higher percent of male, Han-Chinese, retired, unmarried and widowed, more likely to experience hemorrhagic stroke, comorbidities, and intracranial procedure (Table 1). Median annual concentration prior to hospitalization was 47.48 (IQR: 40.45 to 55.59) μg/m^3^ for ambient PM_2.5_, 2.18 (IQR: 1.90 to 2.61) μg/m^3^ for BC, 11.55 (IQR: 10.11 to 13.58) μg/m^3^ for OM, 8.40 (IQR: 7.26 to 10.07) μg/m^3^ for ${SO}_{4}^{2-}$, 10.76 (IQR: 9.05 to 12.35) μg/m^3^ for ${NO}_{3}^{-}$, and 7.23 (IQR: 6.27 to 8.25) μg/m^3^ for ${NH}_{4}^{+}$. PM_2.5_ and its chemical components showed high pairwise correlation (Fig. 4). The median and IQR concentrations of PM_2.5_ mass and its chemical components were higher in Shanxi, followed by those in Sichuan, and Guangxi and Zhanjiang (Fig. S1).

**Table 1:** Ambient PM_2.5_ composition, patient and hospital characteristics in overall sample and by case fatality.

Characteristics	Overall	Case fatality		P-value
	N = 3,069,093	No	Yes	
		(N = 3,037,561 [99%])	31,532 (1.03%)	
**Annual concentrations of PM**_**2.5**_**and its chemical composition prior to hospitalization, μg/m**^**3**^				
PM_2.5_, median [IQR]	47.48 [40.45, 55.59]	47.49 [40.46, 55.61]	46.42 [40.08, 53.87]	<0.001
BC, median [IQR]	2.18 [1.90, 2.61]	2.18 [1.90, 2.61]	2.21 [1.95, 2.58]	<0.001
OM, median [IQR]	11.55 [10.11, 13.58]	11.55 [10.11, 13.59]	11.67 [10.43, 13.48]	<0.001
${SO}_{4}^{2-}$, median [IQR]	8.40 [7.26, 10.07]	8.40 [7.26, 10.07]	8.40 [7.26, 9.85]	<0.001
${NO}_{3}^{-}$, median [IQR]	10.76 [9.05, 12.35]	10.77 [9.05, 12.35]	10.54 [8.80, 12.06]	<0.001
${NH}_{4}^{+}$, median [IQR]	7.23 [6.27, 8.25]	7.23 [6.27, 8.26]	7.15 [6.20, 8.06]	<0.001
**Patient demographics and socioeconomic status**				
Age, y, median [IQR]	68.00 [59.78, 76.00]	68.00 [59.76, 76.00]	73.00 [61.00, 81.36]	<0.001
Sex				
Female	1,329,856 (43.33)	1,318,105 (43.39)	11,751 (37.27)	<0.001
Male	1,739,237 (56.67)	1,719,456 (56.61)	19,781 (62.73)	
Ethnicity				
Han	3,036,016 (98.92)	3,004,780 (98.92)	31,236 (99.06)	0.018
non-Han	33,077 (1.08)	32,781 (1.08)	296 (0.94)	
Occupation				
Public sector	80,702 (2.63)	79,776 (2.63)	926 (2.94)	<0.001
Private sector	180,406 (5.88)	178,323 (5.87)	2083 (6.61)	
Agriculture	1,632,779 (53.20)	1,620,302 (53.34)	12,477 (39.57)	
Unemployed	80,227 (2.61)	79,247 (2.61)	980 (3.11)	
Retired	373,292 (12.16)	367,379 (12.09)	5913 (18.75)	
Other	721,687 (23.51)	712,534 (23.46)	9153 (29.03)	
Marital status				
Married	2,564,883 (83.57)	2,540,390 (83.63)	24,493 (77.68)	<0.001
Unmarried	84,961 (2.77)	82,760 (2.72)	2201 (6.98)	
Widowed	149,403 (4.87)	146,816 (4.83)	2587 (8.20)	
Divorced	38,818 (1.26)	37,696 (1.24)	1122 (3.56)	
Other	231,028 (7.53)	229,899 (7.57)	1129 (3.58)	
**Comorbidities and procedures**				
Stroke subtype				
Hemorrhagic	751,946 (24.50)	734,388 (24.18)	17,558 (55.68)	<0.001
Ischemic	2,180,139 (71.04)	2,167,955 (71.37)	12,184 (38.64)	
Unspecified	137,008 (4.46)	135,218 (4.45)	1790 (5.68)	
Hypertension	1,594,311 (51.95)	1,577,936 (51.95)	16,375 (51.93)	0.959
Diabetes	408,309 (13.30)	403,421 (13.28)	4888 (15.50)	<0.001
Congestive heart failure	105,375 (3.43)	102,201 (3.36)	3174 (10.07)	<0.001
Cardiac arrhythmias	161,259 (5.25)	157,562 (5.19)	3697 (11.72)	<0.001
Peripheral vascular disorders	283,565 (9.24)	282,598 (9.30)	967 (3.07)	<0.001
Liver disease	83,828 (2.73)	82,464 (2.71)	1364 (4.33)	<0.001
Elixhauser comorbidity index, median [IQR]	4.00 [0.00, 4.00]	4.00 [0.00, 4.00]	4.00 [0.00, 8.00]	<0.001
Intracranial procedure	50,871 (1.66)	48,310 (1.59)	2561 (8.12)	<0.001
**Hospital characteristics**				
Hospital level				
Non-Tertiary	1,648,746 (53.72)	1,633,966 (53.79)	14,780 (46.87)	<0.001
Tertiary	1,420,347 (46.28)	1,403,595 (46.21)	16,752 (53.13)	
**Meteorologic variables (7-day average prior to hospitalization)**				
Temperature, °C, median [IQR]	16.20 [8.49, 22.31]	16.20 [8.48, 22.31]	16.09 [9.00, 22.58]	<0.001
Relative humidity, %, median [IQR]	70.40 [55.52, 78.35]	70.35 [55.39, 78.33]	73.89 [65.75, 79.88]	<0.001

PM_2.5_ = ambient particulate matter with diameter ≤2.5 μm; BC = black carbon; OM = organic matter; ${SO}_{4}^{2-}$ = sulphate; ${NO}_{3}^{-}$ = nitrate; ${NH}_{4}^{+}$ = ammonium.

### Associations of PM_2.5_ and its chemical components with in-hospital case fatality

Each IQR increment in annual concentrations of PM_2.5_ and its three chemical components prior to the day of hospitalization was significantly associated with increased risk of fatality (Table 2): the ORs were 1.137 (95% CI: 1.118–1.157, IQR: 15.14 μg/m^3^) for PM_2.5_, 1.108 (95% CI: 1.091–1.126, IQR: 0.71 μg/m^3^) for BC, 1.086 (95% CI: 1.069–1.104, IQR: 3.47 μg/m^3^) for OM, and 1.065 (95% CI: 1.048–1.083, IQR: 2.81 μg/m^3^) for ${SO}_{4}^{2-}$. The associations were insignificant for ${NO}_{3}^{-}$ (OR: 0.991, 95% CI: 0.975–1.008, and IQR: 3.30 μg/m^3^) and significantly negative for ${NH}_{4}^{+}$ (OR: 0.977, 95% CI: 0.962–0.992, and IQR: 1.98 μg/m^3^) (Table 2).

**Table 2:** Associations of interquartile range increment in ambient PM_2.5_ and its chemical composition with in-hospital case fatality in patients with stroke.

Air pollutant	**IQR** (μg/m^3^)	OR (95% CI)	
		Age-adjusted model	Fully-adjusted model
PM_2.5_	15.14	1.261 (1.240–1.281)	1.137 (1.118–1.157)
BC	0.71	1.243 (1.224–1.261)	1.108 (1.091–1.126)
OM	3.47	1.194 (1.176–1.212)	1.086 (1.069–1.104)
${SO}_{4}^{2-}$	2.81	1.160 (1.142–1.179)	1.065 (1.048–1.083)
${NO}_{3}^{-}$	3.3	1.033 (1.016–1.050)	0.991 (0.975–1.008)
${NH}_{4}^{+}$	1.98	0.986 (0.971–1.001)	0.977 (0.962–0.992)

BC = black carbon; CI = confidence interval; IQR = interquartile range; ${NH}_{4}^{+}$ = ammonium; ${NO}_{3}^{-}$ = nitrate; OM = organic matter; OR: odds ratio; PM_2.5_ = particulate matter <2.5 μm in aerodynamic diameter; ${SO}_{4}^{2-}$ = sulphate. Fully-adjusted model accounts for age, sex, ethnicity, occupation, marital status, hypertension, diabetes, congestive heart failure, cardiac arrhythmias, peripheral vascular disorders, liver disease, stroke subtypes, intracranial procedure, hospital level, splines of temperature and relative humidity (five degrees of freedom), and province.

**Fig. 5:**
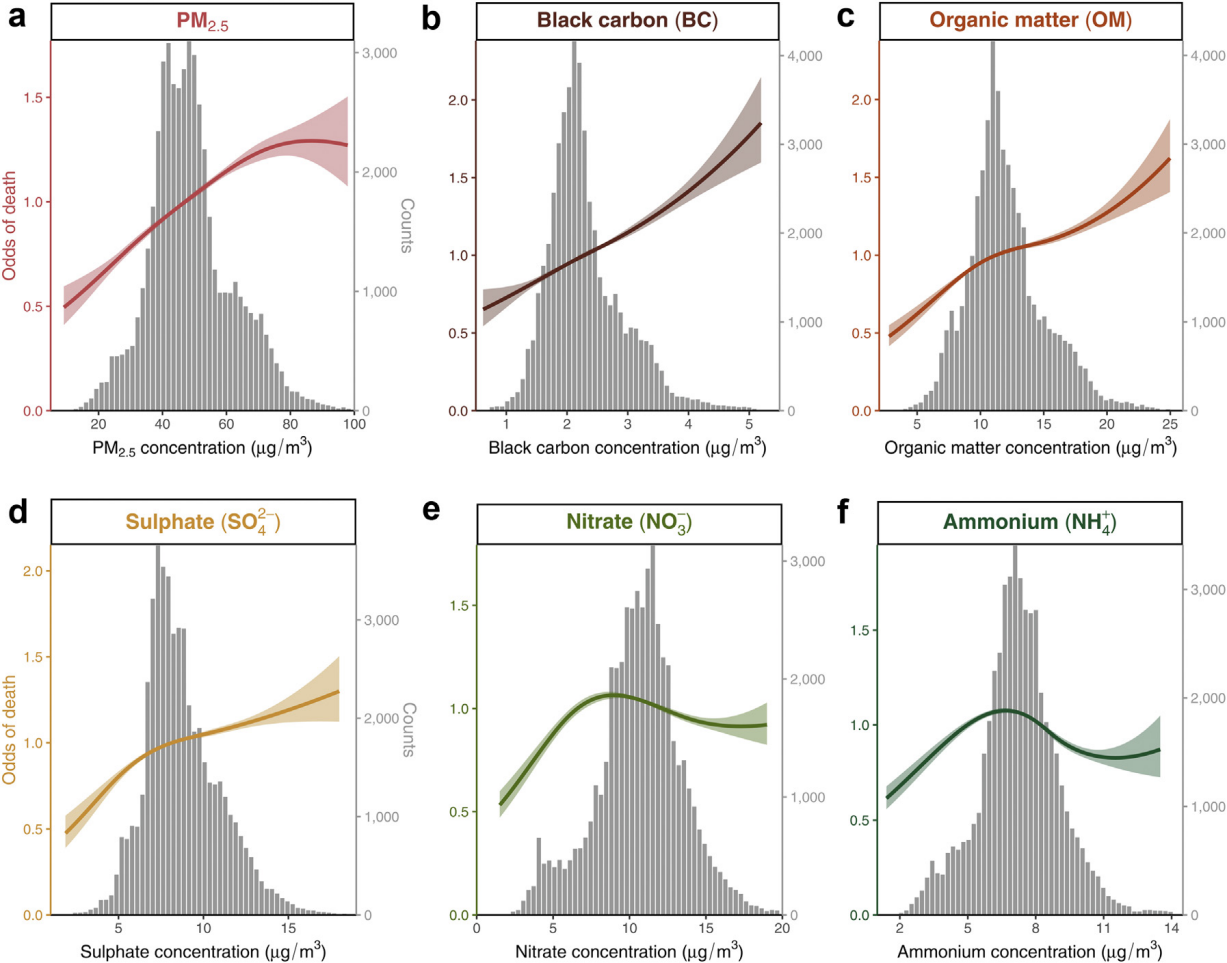
**Exposure-response relationships of annual concentrations of(a)ambient PM**_**2.5**_**and its chemical compositions [(b)black carbon (BC),(c)organic matter (OM),(d)sulphate (**$SO_{4}^{2-}$**),(e)nitrate (**$NO_{3}^{-}$**), and(f)ammonium (**$NH_{4}^{+}$**)] with stroke in-hospital case fatality**. Annual concentrations of PM_2.5_ and its chemical compositions were assessed as 365-day averages prior to the day of hospitalization using 10 ∗ 10 km grids. The solid curves represent the odds of in-hospital fatality, and the shaded bands are the associated 95% confidence intervals; the gray bars are the histogram showing the statistical distribution of PM_2.5_ and its chemical compositions in the study sample. PM_2.5_: particulate matter <2.5 μm in aerodynamic diameter.

Fig. 5 shows the concentration–response relationships of PM_2.5_ and its chemical components with in-hospital fatality using spline analyses. Consistent with the directions of OR estimates, PM_2.5_, BC, OM, and ${SO}_{4}^{2-}$ exhibited monotonically increasing trend for stroke fatality at elevated concentrations of pollution, among which PM_2.5_ and ${SO}_{4}^{2-}$ showed a slight decreasing gradient while BC and OM showed a modestly increasing gradient. The relationships of ${NO}_{3}^{-}$ and ${NH}_{4}^{+}$ with the risk of fatality were increasing at lower concentrations but showed a decreasing trend at higher concentrations.

When stratified by stroke subtypes, the associations of ambient PM_2.5_ and its chemical components with stroke fatality were stronger in patients with ischemic stroke than those with hemorrhagic stroke (Table 3). The ORs and their associated 95% CIs per IQR increment among patients with ischemic stroke were 1.158 (1.130–1.188) for PM_2.5_, 1.141 (1.116–1.167) for BC, 1.113 (1.087–1.139) for OM, and 1.102 (1.075–1.129) for ${SO}_{4}^{2-}$; while the magnitude of associations was smaller among patients with hemorrhagic stroke: 1.082 (1.047–1.119) for PM_2.5_, 1.069 (1.039–1.100) for BC, 1.055 (1.026–1.085) for OM, and 1.020 (0.991–1.049) for ${SO}_{4}^{2-}$.

**Table 3:** Associations of interquartile range increment in ambient PM_2.5_ and its chemical composition with in-hospital case fatality by stroke subtypes.

Air pollutant	Ischemic stroke (n = 2,180,139)			Hemorrhagic stroke (n = 751,946)			Unspecific stroke (n = 137,008)		
	IQR (μg/m^3^)	OR (95% CI)		IQR (μg/m^3^)	OR (95% CI)		IQR (μg/m^3^)	OR (95% CI)	
		Age-adjusted	Fully-adjusted		Age-adjusted	Fully-adjusted		Age-adjusted	Fully-adjusted
PM_2.5_	13.85	1.293 (1.262–1.324)	1.158 (1.130–1.188)	21.6	1.174 (1.137–1.212)	1.082 (1.047–1.119)	14.7	1.339 (1.239–1.448)	1.204 (1.110–1.305)
BC	0.64	1.278 (1.251–1.306)	1.141 (1.116–1.167)	0.93	1.107 (1.077–1.138)	1.069 (1.039–1.100)	0.55	1.224 (1.153–1.301)	1.108 (1.040–1.180)
OM	3.25	1.233 (1.206–1.261)	1.113 (1.087–1.139)	4.55	1.077 (1.048–1.107)	1.055 (1.026–1.085)	3	1.234 (1.154–1.32)	1.123 (1.047–1.205)
${SO}_{4}^{2-}$	2.62	1.201 (1.172–1.229)	1.102 (1.075–1.129)	3.5	1.035 (1.007–1.064)	1.020 (0.991–1.049)	2.11	1.120 (1.058–1.186)	1.054 (0.993–1.119)
${NO}_{3}^{-}$	3.11	1.049 (1.023–1.075)	0.995 (0.970–1.020)	3.88	0.997 (0.975–1.019)	0.975 (0.949–1.001)	3.76	1.186 (1.085–1.296)	1.118 (1.020–1.225)
${NH}_{4}^{+}$	1.87	1.009 (0.985–1.033)	0.984 (0.961–1.008)	2.3	0.937 (0.916–0.959)	0.920 (0.898–0.943)	2.23	1.065 (0.984–1.153)	1.047 (0.963–1.137)

BC = black carbon; CI = confidence interval; IQR = interquartile range; ${NH}_{4}^{+}$ = ammonium; ${NO}_{3}^{-}$ = nitrate; OM = organic matter; OR = odds ratio; PM_2.5_ = particulate matter <2.5 μm in aerodynamic diameter; ${SO}_{4}^{2-}$ = sulphate. Fully-adjusted models account for age, sex, ethnicity, occupation, marital status, hypertension, diabetes, congestive heart failure, cardiac arrhythmias, peripheral vascular disorders, liver disease, intracranial procedure, hospital level, splines of temperature and relative humidity (five degrees of freedom), and province.

### Population attributable fractions

**Fig. 6:**
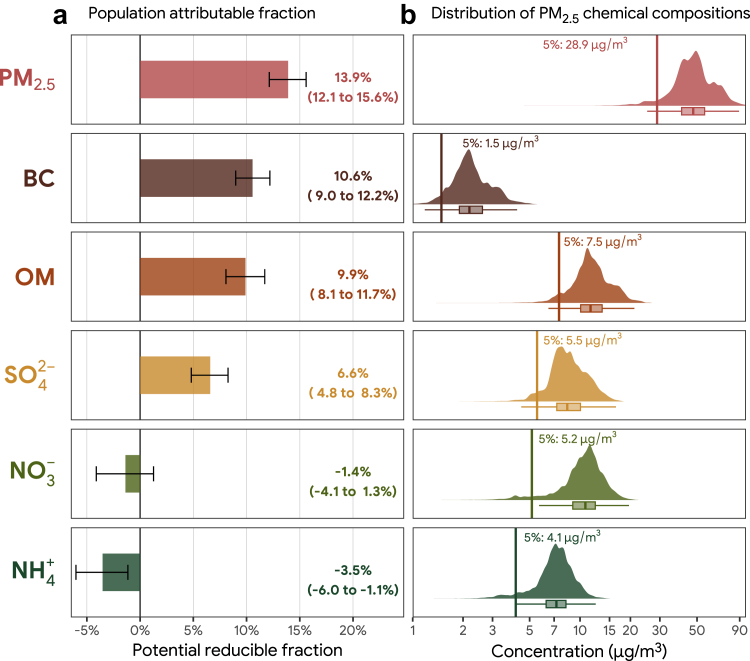
**(a)Population attributable fraction of in-hospital case fatality attributable to ambient PM**_**2.5**_**and its chemical compositions [black carbon (BC), organic matter (OM), sulphate (**$SO_{4}^{2-}$**), nitrate (**$NO_{3}^{-}$**), and ammonium (**$NH_{4}^{+}$**)] in patients with strokeand (b) the statistical distributions and the fifth percentiles of PM_2.5_ and its chemical compositions**.

Using counterfactual analyses, we further estimated the PAFs of PM_2.5_ and its chemical components to fatality (Fig. 6), which is the proportional reduction in fatality that would occur if the concentrations of ambient PM_2.5_ and its chemical components were reduced to the 5^th^ percentiles in their statistical distributions. The PAFs were 10.6% (95% CI: 9.1–12.2%) for BC (median: 2.18 μg/m^3^, 5^th^ percentile: 1.5 μg/m^3^), 9.9% (95% CI: 8.2–11.7%) for OM (median: 11.55 μg/m^3^, 5^th^ percentile: 7.5 μg/m^3^), and 6.6% (95% CI: 4.9–8.3%) for ${SO}_{4}^{2-}$ (median: 8.40 μg/m^3^, 5^th^ percentile: 5.5 μg/m^3^). The pattern of PAF for each PM_2.5_ chemical components to fatality is consistent with that of ORs.

### Sensitivity analyses

In Cox proportional hazard models where time to fatality was taken as the outcome, although the estimates were smaller in Cox models, the pattern of the associations was consistent with that in main models (hazard ratios: PM_2.5_ > BC > OM > ${SO}_{4}^{2-}$) (Table S1). When Elixhauser comorbidity score of 30 comorbidities was included in the models, the magnitude of ORs was attenuated, but the pattern of associations was stable (Table S2). In Table S3, the E-values for ORs of PM_2.5_, BC, OM, and ${SO}_{4}^{2-}$ were >1.3, indicating relatively strong evidence for causality, while the E-values were 1.179 for OR and 1.098 for 95% CI of ${NH}_{4}^{+}$, suggesting slightly weaker evidence for causality. In a sample with informative occupation and marital status (N = 2,300,657, 75% of the full sample), the OR estimates were slightly smaller but consistent in the pattern (Table S4). In models conducted in each province, the ORs estimated in Guangxi and Zhanjiang were larger than those in Shanxi and Sichuan, while the trend of risk ratio estimates remained consistent (Table S5). In a sample with unified time range from 2013 to 2016, the results were consistent with the main findings with slightly larger effect sizes while the ORs for ${NO}_{3}^{-}$ and ${NH}_{4}^{+}$ were insignificant (Table S6). In patients without previous admissions, we found that the effect sizes were smaller compared to those of full sample in Sichuan, but pattern of effect sizes was the same: BC showed the highest risk OR, followed by OM and ${SO}_{4}^{2-}$.

## Discussion

In our analysis of a large multiprovince sample of over 3 million hospitalized records in China, we found that the annual concentrations of three PM_2.5_ chemical components were significantly associated with the in-hospital fatality of stroke: BC showed the highest risk ratio per IQR increment, followed by OM and ${SO}_{4}^{2-}$. Counterfactual analyses revealed the same trend of PAFs. The results were consistent in a set of sensitivity analyses including alternating the statistical models and comorbidity covariate sets, computing E-values to measure the strength of causality, and estimating results by provinces and on data with informative occupation and marital status.

BC is exclusively derived from incomplete combustion in vehicular traffic, residential biofuel burning, and industrial emissions.^30^^,^^31^ OM is primarily produced by a mixture of combustion-related emissions and secondary reactions including biogenic volatile organic compounds. ${SO}_{4}^{2-}$ is a secondary inorganic ion produced by anthropogenic fossil fuel combustion and natural sources (sulfur-containing gases from oceans and volcanos).^32^^,^^33^ Our results support the deleterious role of combustion particles from anthropogenic emission sources (BC, OM, and ${SO}_{4}^{2-}$) on stroke mortality. The adverse health burden attributable to BC and OM is further compounded by their roles in supercharging heat-absorbing greenhouse effects and precipitating global warming effects in a manner similar to carbon dioxide,^31^^,^^34^ which exerts an additional burden on human health. Therefore, our study highlights the need to promulgate finer and more tailored guidelines for PM chemical components,^35^ make strategic plans to curb the emissions of most hazardous chemical components such as improving combustion efficiency and adopting green-energy vehicles.

Several biological mechanisms may explain these observed associations, of which oxidative stress is the most common mechanism. Animal- and human-based studies have indicated that BC, OM, and ${SO}_{4}^{2-}$ can induce oxidative stress, vascular inflammation responses, as well as other physiological processes involving coagulation and thrombosis, after PM particles penetrate lung tissues and enter the blood circulation.^36^, ^37^, ^38^, ^39^ This mechanism is also in line with our epidemiological findings that the associations between PM chemical components and stroke fatality are stronger in patients with ischemic stroke than in those with hemorrhagic stroke. Ischemic stroke accounts for most stroke subtypes, and it is caused by blood clots and thrombosis, while hemorrhagic stroke is the consequence of blood vessel burst. Animal-based studies have suggested that PM_2.5_ is associated with atherosclerosis progression via mechanisms including systemic inflammation and the formation of reactive oxygen species, which are more specific to ischemic stroke and subsequent outcomes.^5^^,^^40^ Other potential biological mechanisms include vascular endothelial dysfunction^38^^,^^40^^,^^41^ and an altered autonomic nervous system balance.^42^

Our findings are in line with those of a few other studies. Another nationwide cohort study of 90,672 Chinese adults (mean age 46.2 years and 56.6% female)^43^ reported similarly deleterious associations for BC [hazard ratio and 95% CI: 1.19 (1.07–1.38)], OM [1.15 (1.05, 1.30)], and ${SO}_{4}^{2-}$ [1.14 (1.01, 1.34)], but their study reported positive signals for ${NO}_{3}^{-}$ and ${NH}_{4}^{+}$. Compared with their results, our risk ratio estimates were slightly larger, likely because our sample included hospitalized patients who were older and sicker and may therefore be more susceptible to PM_2.5_. Another individual-level analysis conducted in Europe has also reported that BC or its surrogate measures (PM absorbance) were associated with increased risk of incident stroke [HR: 1.041 (95% CI: 1.004–1.08) per IQR increment].^44^ Other ecological studies that used a time-series framework and were based on Chinese data reported much smaller risk ratios for BC or OM, and the excessive risks ranged from 1.67% to 2.83%.^45^^,^^46^

Our finding on null association between ${NO}_{3}^{-}$ and stroke fatality is consistent with most epidemiological and toxicological data that suggest little to none effects of nitrate on health effects at ambient levels.^33^^,^^47^^,^^48^ Population-based studies suggest that ${NO}_{3}^{-}$ is not likely to pose a significant health risk by itself,^48^ while toxicological studies use nitrate concentrations much higher than ambient level and the conclusions provide little practical insights to real-world human health effects.^33^ The slight protective while significant estimates for ${NH}_{4}^{+}$ in this study may not be interpreted as truly protective. The concentration-response curve depicted an “inverse-U” relationship, indicating a negative association at higher spectrum of ambient ${NH}_{4}^{+}$. This is partially supported by our results stratified by provinces, in which the estimates were protective in Sichuan and Shanxi (higher levels of ${NH}_{4}^{+}$), but deleterious in Guangxi and Guangdong (lower levels of ${NH}_{4}^{+}$). Further external data are needed to better understand the negative associations between ${NH}_{4}^{+}$ at higher levels and stroke mortality.

### Limitations and strengths

Our study should be interpreted in view of several limitations. We cannot rule out potential confounders such as cigarette smoking, stroke severity, and physical activity in this study based on claims data, but the potential biases may have been mitigated since their proxy variables (such as age, sex, occupation) have been included in the regression models. The results were based on data from four provinces in China and have limited generalizability to other provinces or other developing countries; data from Zhanjiang city were chosen as a result of data availability and may not be representative of Guangdong province. The TAP datasets essentially measure the concentrations of outdoor air pollution, so we were not able to measure the concentrations of indoor PM_2.5_ chemical composition. This limitation is mitigated by the fact that inpatient departments in Chinese hospitals have a high adoption rate of central air conditioner units that considerably reduces air pollution. Noise and air pollution share many sources and are often co-localized in urban areas, but we were not able to account for it in the models due to the lack of data. We were not able to investigate the potential interactions across multiple PM_2.5_ chemical components due to the high correlation between constituents and a lack of appropriate multi-constituent statistical models.^49^ Since only one residential address was collected, patients who moved during the exposure measurement period may cause exposure misclassification, but the proportion was likely small since all the included patients had stroke and had limited ability to move extensively. Although we calculated the E-values to assess evidence for causality, this is an observational study and cannot quantify the true causal effects. Although there are many animal-based studies investigated the biological mechanisms of the observed associations between PM_2.5_ and stroke, the mechanisms for the chemical components of PM_2.5_ are not clear and needs further studies.^50^ The patients were nested within hospitals and random-effect models may be applied at hospital-level, but we were unable to fit these models due to the large sample size and model complexity.

Despite these limitations, our study highlights several strengths. Our study included a large sample size of over 3 million hospitalized records with stroke spanning four provinces in China, yielding a large statistical power and precise uncertainty estimates. Most of the evidence on association between air pollution and health outcomes was derived from in Europe and North America, with low concentrations of PM_2.5_ and its chemical components. In contrast, this study is conducted in a fast-developing country with both looming burden of stroke mortality and exceedingly high concentrations of air pollution. The characterization of relationship between PM_2.5_ chemical components and stroke mortality at higher spectrum of air pollution may be more useful to a cascade of LMICs that face similar challenges such as India. The exposure data in our study had a high temporal (daily) and spatial (10 ∗ 10 km grids) resolution, together with individual-level residential addresses, produced accurate exposure measurement and valid risk ratio estimates.

### Conclusions

In summary, using data from approximately three million hospitalizations in China, we found that BC, OM, and ${SO}_{4}^{2-}$ were significantly associated with the highest risk of stroke fatality. Our results suggest the need to develop guidelines for PM chemical components and curb the emission of the most hazardous chemical components to improve the health outcomes of patients hospitalized with stroke.

## Contributors

Dr. Jay Pan and Dr. Hualiang Lin had full access to all of the data in the study and takes responsibility for the integrity of the data and the accuracy of the data analysis.

*Concept and design*: Cai, Lin, X., Pan, Lin, H.

*Acquisition, analysis, or interpretation of data*: Cai, Lin, X., Wang, Pan, Lin, H.

*Drafting of the manuscript*: Cai, Lin, X., Lin, H.

*Critical revision of the manuscript for important intellectual content*: All authors.

*Statistical analysis*: Cai, Lin, X., Zhang, S.

Obtained funding: Lin, H.

Administrative, technical, or material support: Pan, Lin, H.

Supervision: Pan, Lin, H.

## Data sharing statement

The authors do not have permission to share the data used in this study.

## Editor note

The Lancet Group takes a neutral position with respect to territorial claims in published maps and institutional affiliations.

## Declaration of interests

The authors declare no competing interests.

## Appendix A Supplementary data

Caption for supplementary materials
