# Supplementary material for: Long-term exposure to ambient fine particulate matter chemical composition and in-hospital case fatality among patients with stroke in China
Source: Lancet Reg Health West Pac. 2023 Jan 13;32:100679. doi: 10.1016/j.lanwpc.2022.100679 (PMC9918804; doi:10.1016/j.lanwpc.2022.100679)
Supplement: Caption for supplementary materials [file mmc1.docx]

**Caption for supplementary materials**

**Table S1** Hazard ratios (95% confidence intervals) per interquartile range increment in ambient PM_2.5_ and its chemical composition with in-hospital case fatality using Cox proportional hazard models.

**Table S2**. Odds ratios and 95% confidence intervals per interquartile increment on the associations of ambient PM_2.5_ and its chemical components with the risk of in-hospital case fatality using Elixhauser comorbidity score as covariate.

**Table S3**. E-values for the odds ratios and their 95% confidence intervals per interquartile increment on the associations of ambient PM_2.5_ and its chemical components with the risk of in-hospital case fatality.

**Table S4**. Odds ratios and 95% confidence intervals per interquartile increment on the associations of ambient PM_2.5_ and its chemical components with the risk of in-hospital case fatality among patients with informative occupation and marital status.

**Table S5** Odds ratios and 95% confidence intervals per interquartile range increment in ambient PM_2.5_ and its chemical composition with fatality by province.

**Table S6**. Odds ratios and 95% confidence intervals per interquartile increment on the associations of ambient PM_2.5_ and its chemical components with the risk of in-hospital case fatality by restricting to a unified time range of from 2013 to 2016 (N=825,340).

**Table S7** Odds ratios and 95% confidence intervals per interquartile increment on the associations of ambient PM_2.5_ and its chemical components with the risk of in-hospital case fatality based on sample without previous admissions (N=1,006,208) and all samples (N=1,414,898) in Sichuan.

**Figure S1.** Boxplots and kernel density plots of PM_2.5_ mass and its chemical components by province. Zhanjiang city was included as a part of Guangxi since it has relatively small sample size and was contiguous with Guangxi.
