# Supplementary material for: Long-term exposure to ambient fine particulate matter chemical composition and in-hospital case fatality among patients with stroke in China
Source: Lancet Reg Health West Pac. 2023 Jan 13;32:100679. doi: 10.1016/j.lanwpc.2022.100679 (PMC9918804; doi:10.1016/j.lanwpc.2022.100679)
Supplement: Supplementary Tables S1–S7 and Fig. S1 [file mmc2.docx]

**Supplementary materials**

**Long-term exposure to ambient fine particulate matter chemical composition and in-hospital case fatality among patients with stroke in China**

**Table of Contents**

| **Caption** | **Page** |
| --- | --- |
| **Table S1** Hazard ratios (95% confidence intervals) per interquartile range increment in ambient PM_2.5_ and its chemical composition with in-hospital case fatality using Cox proportional hazard models. | 2 |
| **Table S2**. Odds ratios and 95% confidence intervals per interquartile increment on the associations of ambient PM_2.5_ and its chemical components with the risk of in-hospital case fatality using Elixhauser comorbidity score as covariate. | 2 |
| **Table S3**. E-values for the odds ratios and their 95% confidence intervals per interquartile increment on the associations of ambient PM_2.5_ and its chemical components with the risk of in-hospital case fatality. | 3 |
| **Table S4**. Odds ratios and 95% confidence intervals per interquartile increment on the associations of ambient PM_2.5_ and its chemical components with the risk of in-hospital case fatality among patients with informative occupation and marital status. | 3 |
| **Table S5** Odds ratios and 95% confidence intervals per interquartile range increment in ambient PM_2.5_ and its chemical composition with fatality by province. | 4 |
| **Table S6**. Odds ratios and 95% confidence intervals per interquartile increment on the associations of ambient PM_2.5_ and its chemical components with the risk of in-hospital case fatality by restricting to a unified time range of from 2013 to 2016 (N=825,340). | 5 |
| **Table S7** Odds ratios and 95% confidence intervals per interquartile increment on the associations of ambient PM_2.5_ and its chemical components with the risk of in-hospital case fatality based on sample without previous admissions (N=1,006,208) and all samples (N=1,414,898) in Sichuan. | 5 |
| **Figure S1.** Boxplots and kernel density plots of PM_2.5_ mass and its chemical components by province. Zhanjiang city was included as a part of Guangxi since it has relatively small sample size and was contiguous with Guangxi. | 6 |

| **Table S1** Hazard ratios (95% confidence intervals) per interquartile range increment in ambient PM_2.5_ and its chemical composition with in-hospital case fatality using Cox proportional hazard models | | | |
| --- | --- | --- | --- |
| **Air pollutants** | **IQR (µg/m^3^)** | **HR (95% CI) *** | **P-value *** |
| PM_2.5_ | 15.14 | 1.102 (1.084-1.121) | <0.001 |
| BC | 0.71 | 1.077 (1.061-1.094) | <0.001 |
| OM | 3.47 | 1.065 (1.049-1.081) | <0.001 |
| $\mathrm{SO}_{4}^{2-}$ | 2.81 | 1.032 (1.015-1.049) | <0.001 |
| $\mathrm{NO}_{3}^{-}$ | 3.30 | 0.98 (0.964-0.996) | 0.013 |
| $\mathrm{NH}_{4}^{+}$ | 1.98 | 0.947 (0.933-0.962) | <0.001 |

Abbreviations: BC=black carbon; CI: confidence interval; IQR: interquartile range; $\mathrm{NH}_{4}^{+}$= ammonium; $\mathrm{NO}_{3}^{-}$= nitrate; OM=organic matter; HR: hazard ratio; PM_2.5_: PM_2.5_=particulate matter <2.5 µm in aerodynamic diameter; $\mathrm{SO}_{4}^{2-}$=sulphate.
* Models adjusted for age, sex, ethnicity, occupation, marital status, hypertension, diabetes, congestive heart failure, cardiac arrhythmias, peripheral vascular disorders, liver disease, stroke subtypes, intracranial procedure, hospital level, splines of temperature and relative humidity (five degrees of freedom), and province.

| **Table S2**. Odds ratios and 95% confidence intervals per interquartile increment on the associations of ambient PM_2.5_ and its chemical components with the risk of in-hospital case fatality using Elixhauser comorbidity score as covariate | | | |
| --- | --- | --- | --- |
| **Air pollutants** | **IQR (μg/m^3^)** | **OR (95% CI) *** | **P-value *** |
| PM_2.5_ | 15.14 | 1.103 (1.084-1.122) | <0.001 |
| BC | 0.71 | 1.071 (1.054-1.088) | <0.001 |
| OM | 3.47 | 1.052 (1.035-1.069) | <0.001 |
| $\mathrm{SO}_{4}^{2-}$ | 2.81 | 1.048 (1.031-1.066) | <0.001 |
| $\mathrm{NO}_{3}^{-}$ | 3.3 | 0.972 (0.956-0.988) | <0.001 |
| $\mathrm{NH}_{4}^{+}$ | 1.98 | 0.953 (0.939-0.969) | <0.001 |
| Abbreviations: BC=black carbon; CI: confidence interval; IQR: interquartile range; $\mathrm{NH}_{4}^{+}$= ammonium; $\mathrm{NO}_{3}^{-}$= nitrate; OM=organic matter; OR: odds ratio; PM_2.5_: PM_2.5_=particulate matter <2.5 µm in aerodynamic diameter; $\mathrm{SO}_{4}^{2-}$=sulphate. *Models adjusted for age, sex, ethnicity, occupation, marital status, Elixhauser comorbidity score, stroke subtypes, intracranial procedure, hospital level, splines of temperature and relative humidity (five degrees of freedom), and province. | | | |

| **Table S3**. E-values for the odds ratios and their 95% confidence intervals per interquartile increment on the associations of ambient PM_2.5_ and its chemical components with the risk of in-hospital case fatality | | | |
| --- | --- | --- | --- |
| **Air pollutants** | **OR (95% CI)** | **E-value for OR*** | **E-value for 95% CI*** |
| PM_2.5_ | 1.137 (1.118-1.157) | 1.532 | 1.481 |
| BC | 1.108 (1.091-1.126) | 1.453 | 1.406 |
| OM | 1.086 (1.069-1.104) | 1.392 | 1.341 |
| $\mathrm{SO}_{4}^{2-}$ | 1.065 (1.048-1.083) | 1.328 | 1.272 |
| $\mathrm{NO}_{3}^{-}$ | 0.991 (0.975-1.008) | 1.105 | 1.000 |
| $\mathrm{NH}_{4}^{+}$ | 0.977 (0.962-0.992) | 1.179 | 1.098 |
| Abbreviations: BC=black carbon; CI: confidence interval; IQR: interquartile range; $\mathrm{NH}_{4}^{+}$= ammonium; $\mathrm{NO}_{3}^{-}$= nitrate; OM=organic matter; OR: odds ratio; PM_2.5_: PM_2.5_=particulate matter <2.5 µm in aerodynamic diameter; $\mathrm{SO}_{4}^{2-}$=sulphate.  * The E-values were computed for the scenarios of odds ratios with the prevalence of outcomes < 15% (rare events). | | | |

| **Table S4**. Odds ratios and 95% confidence intervals per interquartile increment on the associations of ambient PM_2.5_ and its chemical components with the risk of in-hospital case fatality among patients with informative occupation and marital status | | | |
| --- | --- | --- | --- |
| **Air pollutants** | **IQR (μg/m^3^)** | **OR (95% CI) *** | **P-value *** |
| PM_2.5_ | 16.58 | 1.139 (1.113-1.164) | <0.001 |
| BC | 0.76 | 1.099 (1.078-1.122) | <0.002 |
| OM | 3.65 | 1.075 (1.054-1.096) | <0.003 |
| $\mathrm{SO}_{4}^{2-}$ | 3.02 | 1.060 (1.038-1.083) | <0.004 |
| $\mathrm{NO}_{3}^{-}$ | 3.50 | 0.987 (0.966-1.008) | 0.223 |
| $\mathrm{NH}_{4}^{+}$ | 2.12 | 0.969 (0.950-0.988) | 0.002 |
| Abbreviations: BC=black carbon; CI: confidence interval; IQR: interquartile range; $\mathrm{NH}_{4}^{+}$= ammonium; $\mathrm{NO}_{3}^{-}$= nitrate; OM=organic matter; HR: hazard ratio; PM_2.5_: PM_2.5_=particulate matter <2.5 µm in aerodynamic diameter; $\mathrm{SO}_{4}^{2-}$=sulphate. * Models adjusted for age, sex, ethnicity, occupation, marital status, hypertension, diabetes, congestive heart failure, cardiac arrhythmias, peripheral vascular disorders, liver disease, stroke subtypes, intracranial procedure, hospital level, splines of temperature and relative humidity (five degrees of freedom), and province. | | | |

| **Table S5** Odds ratios and 95% confidence intervals per interquartile range increment in ambient PM_2.5_ and its chemical composition with fatality by province | | | | | | | | | |
| --- | --- | --- | --- | --- | --- | --- | --- | --- | --- |
| **Air pollutants** | **Sichuan** | | | **Shanxi** | | | **Guangxi and Zhanjiang** | | |
|  | Median (μg/m^3^) | IQR (μg/m^3^) | OR (95% CI) * | Median (μg/m^3^) | IQR (μg/m^3^) | ## * | Median (μg/m^3^) | IQR (μg/m^3^) | OR (95% CI) * |
| PM_2.5_ | 45.09 | 10.24 | 1.105 (1.088-1.123) | 53.12 | 19.31 | 1.143 (1.097-1.191) | 33.07 | 11.79 | 1.255 (1.196-1.317) |
| BC | 2.17 | 0.48 | 1.086 (1.068-1.103) | 2.27 | 1.19 | 1.116 (1.070-1.165) | 1.92 | 0.54 | 1.157 (1.101-1.215) |
| OM | 11.75 | 2.37 | 1.074 (1.057-1.091) | 12.01 | 5.87 | 1.091 (1.044-1.141) | 9.15 | 2.75 | 1.185 (1.134-1.238) |
| $\mathrm{SO}_{4}^{2-}$ | 8.35 | 2.24 | 1.065 (1.046-1.084) | 8.9 | 3.72 | 1.023 (0.980-1.068) | 6.85 | 2.22 | 1.230 (1.172-1.291) |
| $\mathrm{NO}_{3}^{-}$ | 10.81 | 2.43 | 0.988 (0.973-1.003) | 11.42 | 3.49 | 1.009 (0.970-1.050) | 6.01 | 2.53 | 1.231 (1.167-1.299) |
| $\mathrm{NH}_{4}^{+}$ | 7.38 | 1.50 | 0.967 (0.953-0.982) | 7.43 | 2.31 | 0.969 (0.931-1.008) | 4.75 | 1.78 | 1.197 (1.138-1.259) |

Abbreviations: BC=black carbon; CI: confidence interval; IQR: interquartile range; $\mathrm{NH}_{4}^{+}$= ammonium; $\mathrm{NO}_{3}^{-}$= nitrate; OM=organic matter; OR: odds ratio; PM_2.5_: PM_2.5_=particulate matter <2.5 µm in aerodynamic diameter; $\mathrm{SO}_{4}^{2-}$=sulphate.
* Models adjusted for age, sex, ethnicity, occupation, marital status, hypertension, diabetes, congestive heart failure, cardiac arrhythmias, peripheral vascular disorders, liver disease, stroke subtypes, intracranial procedure, hospital level, splines of temperature and relative humidity (five degrees of freedom).

| **Table S6**. Odds ratios and 95% confidence intervals per interquartile increment on the associations of ambient PM_2.5_ and its chemical components with the risk of in-hospital case fatality by restricting to a unified time range of from 2013 to 2016 (N=825,340) | | | |
| --- | --- | --- | --- |
| **Air pollutants** | **IQR (μg/m^3^)** | **OR (95% CI) *** | **P-value *** |
| PM_2.5_ | 16.18 | 1.204 (1.161-1.248) | <0.001 |
| BC | 0.83 | 1.156 (1.118-1.196) | <0.001 |
| OM | 3.97 | 1.135 (1.097-1.174) | <0.001 |
| $\mathrm{SO}_{4}^{2-}$ | 2.81 | 1.108 (1.071-1.147) | <0.001 |
| $\mathrm{NO}_{3}^{-}$ | 3.7 | 1.010 (0.971-1.051) | 0.627 |
| $\mathrm{NH}_{4}^{+}$ | 2.15 | 0.987 (0.953-1.023) | 0.479 |
| Abbreviations: BC=black carbon; CI: confidence interval; IQR: interquartile range; $\mathrm{NH}_{4}^{+}$= ammonium; $\mathrm{NO}_{3}^{-}$= nitrate; OM=organic matter; OR: odds ratio; PM_2.5_: PM_2.5_=particulate matter <2.5 µm in aerodynamic diameter; $\mathrm{SO}_{4}^{2-}$=sulphate. * Models adjusted for age, sex, ethnicity, occupation, marital status, hypertension, diabetes, congestive heart failure, cardiac arrhythmias, peripheral vascular disorders, liver disease, stroke subtypes, intracranial procedure, hospital level, splines of temperature and relative humidity (five degrees of freedom), and province. | | | |

**Table S7** Odds ratios and 95% confidence intervals per interquartile increment on the associations of ambient PM_2.5_ and its chemical components with the risk of in-hospital case fatality based on sample without previous admissions (N=1,006,208) and all samples (N=1,414,898) in Sichuan.

| **Air**  **pollutants** | **Without previous admissions (N=1,006,208)** | | **All sample (N=1,414,898)** | |
| --- | --- | --- | --- | --- |
|  | IQR (µg/m^3^) | OR (95% CI) | IQR (µg/m^3^) | OR (95% CI) |
| PM_2.5_ | 10.59 | 1.072 (1.052-1.092) | 10.24 | 1.105 (1.088-1.123) |
| BC | 0.49 | 1.068 (1.048-1.088) | 0.48 | 1.086 (1.068-1.103) |
| OM | 2.43 | 1.058 (1.039-1.078) | 2.37 | 1.074 (1.057-1.091) |
| $\mathrm{SO}_{4}^{2-}$ | 2.32 | 1.04 (1.018-1.062) | 2.24 | 1.065 (1.046-1.084) |
| $\mathrm{NO}_{3}^{-}$ | 2.53 | 0.982 (0.964-1.000) | 2.43 | 0.988 (0.973-1.003) |
| $\mathrm{NH}_{4}^{+}$ | 1.57 | 0.96 (0.943-0.978) | 1.5 | 0.967 (0.953-0.982) |

Abbreviations: BC=black carbon; CI: confidence interval; IQR: interquartile range; $\mathrm{NH}_{4}^{+}$= ammonium; $\mathrm{NO}_{3}^{-}$= nitrate; OM=organic matter; OR: odds ratio; PM_2.5_: PM_2.5_=particulate matter <2.5 µm in aerodynamic diameter; $\mathrm{SO}_{4}^{2-}$=sulphate.
* Models adjusted for age, sex, ethnicity, occupation, marital status, hypertension, diabetes, congestive heart failure, cardiac arrhythmias, peripheral vascular disorders, liver disease, stroke subtypes, intracranial procedure, hospital level, splines of temperature and relative humidity (five degrees of freedom), and province.

**Figure S1.** Boxplots and kernel density plots of PM_2.5_ mass and its chemical components by province. Zhanjiang city was included as a part of Guangxi since it has relatively small sample size and was contiguous with Guangxi.

**
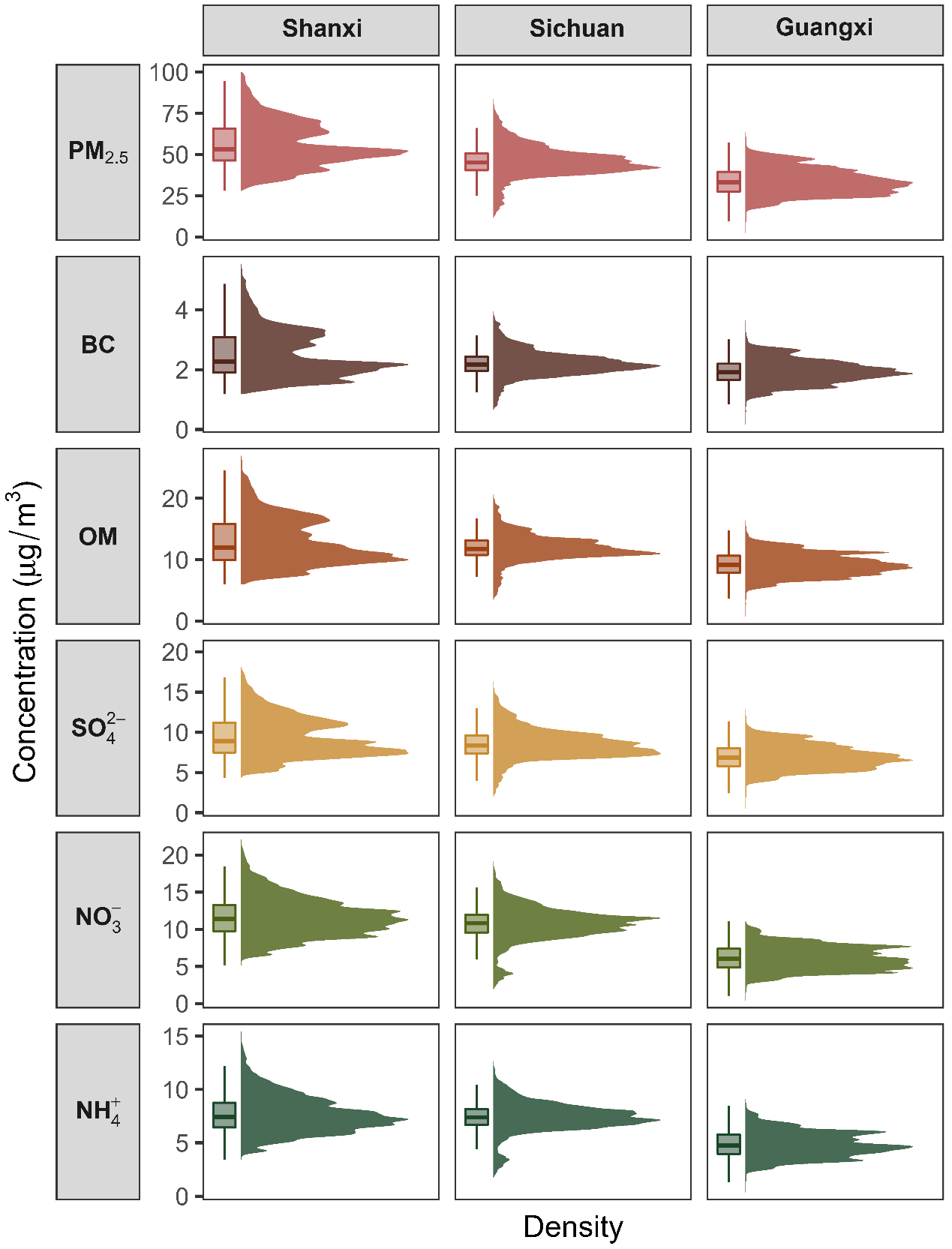
**
